# Microbiological profiling and knowledge of food preservation technology to support guidance on a neutropenic diet for immunocompromised patients

**DOI:** 10.3389/fmicb.2023.1136887

**Published:** 2023-05-25

**Authors:** Thomas De Bock, Liesbeth Jacxsens, Femke Maes, Svenya Van Meerhaeghe, Marina Reygaerts, Mieke Uyttendaele

**Affiliations:** ^1^Research Unit Food Microbiology and Food Preservation, Department of Food Technology, Safety and Health, Faculty of Bioscience Engineering, Ghent University, Ghent, Belgium; ^2^Center for Pediatric Haemato-Oncology and Stem Cell Transplantation, Ghent University Hospital, Ghent, Belgium; ^3^Center for Thoracic Oncology, Ghent University Hospital, Ghent, Belgium

**Keywords:** microbiological quality, food safety, neutropenic diet, immunocompromised, opportunistic pathogens

## Abstract

The current society consists of an increasing number of people vulnerable to infections. For certain people with severe immunodeficiency, a neutropenic or low-microbial diet is being prescribed, which substitutes high-risk foods that are more likely to contain human (opportunistic) pathogens with lower-risk alternatives. These neutropenic dietary guidelines are typically set up from a clinical and nutritional perspective, rather than from a food processing and food preservation perspective. In this study, the current guidelines in use by the Ghent University Hospital were evaluated based on the current knowledge of food processing and preservation technologies and the scientific evidence on microbiological quality, safety, and hygiene of processed foods. Three criteria are identified to be important: (1) the microbial contamination level and composition; (2) the potential presence of established foodborne pathogens such as *Salmonella* spp. (to which a zero-tolerance policy is recommended); and (3) an increased vigilance for *L. monocytogenes* as an opportunistic foodborne pathogen with a high mortality rate in immunocompromised individuals (to which a zero-tolerance policy should apply). A combination of these three criteria was used as a framework for the evaluation of the suitability of foodstuffs to be included in a low-microbial diet. Differences in processing technologies, initial contamination of products, etc., however, lead to a high degree of variability in microbial contamination and make it difficult to unambiguously accept or reject a certain type of foodstuff without prior knowledge of the ingredients and the processing and preservation technologies applied during manufacturing and subsequent storage conditions. A restricted screening on a selection of (minimally processed) plant-based foodstuffs on the retail market in Flanders, Belgium supported decision-making on the inclusion of these food types in a low-microbial diet. Still, when determining the suitability of a foodstuff to be included in a low-microbial diet, not only the microbiological status but also nutritional and sensorial properties should be assessed, which requires multidisciplinary communication and collaboration.

## 1. Introduction

Microorganisms are inherently present in food, although the numbers and types of microorganisms may vary depending upon the type and origin of the food, the hygienic practices upon production, processing, and storage, and the food preservation technology applied. Most microorganisms are harmless, many are highly beneficial, some indicate inappropriate handling or cause spoilage, and a few may cause disease.

Several host factors can contribute to the symptoms and severity of foodborne disease, including primary or secondary immunodeficiency, pregnancy, and age. Accordingly, the sensitive population group is recalled as YOPI (Young, Old, Pregnant, and Immunocompromised; Lund, 2019). There is a wide range in the extent of susceptibility of the different vulnerable groups. For certain people with a severe, established immunodeficiency, such as cancer patients receiving chemotherapy resulting in neutropenia, patients undergoing immunosuppressive therapy during transplantation, etc., a neutropenic or low-microbial diet is often prescribed to prevent foodborne infection and infection-related mortality (Lund, 2014). This diet typically substitutes high-risk foods that are more likely to be a vector for (pathogenic) microorganisms with lower-risk alternatives (Lund and O’Brien, 2011; van Dalen et al., 2016; Lund, 2019). The initial concept of this diet was not only to prevent exposure to established foodborne infectious agents such as *Salmonella* spp., *Campylobacter* spp., etc. but also in general to reduce the intake of (pathogenic) microorganisms in food and as such decrease the risk for (opportunistic) infections in neutropenic patients associated with bacterial translocation from the gut to the bloodstream (Moody et al., 2018; Heng et al., 2020). The value of such a neutropenic diet in reducing (opportunistic) infections in immunosuppressed individuals has, however, been questioned in multiple systematic reviews and meta-analyses (van Dalen et al., 2016; Moody et al., 2018; Ball et al., 2019; Sonbol et al., 2019; Taggart et al., 2019; Heng et al., 2020; Ramamoorthy et al., 2020). The general conclusion being made is that there is no evidence that a neutropenic diet affects infections and related outcomes and it is, therefore, argued that the use of a neutropenic diet should be abandoned and replaced by general safe food handling. It should be stressed, however, as van Dalen et al. (2016) concludes, that although “no evidence of effect” of these diets could be identified, these studies are unable to show “evidence of no effect”. Additional high-quality research is needed to assess the value of the neutropenic diet to prevent infection in patients with neutropenia.

A neutropenic diet typically consists of two parts. On the one hand, guidelines are composed in which, for different food groups, it is indicated which foodstuffs are allowed to be consumed by neutropenic patients or should be avoided. On the other hand, best practices on hygienic measures for the preparation and storage of meals are provided. The composition of a neutropenic diet is, however, not standardized and highly depends on the institution or hospital, although in general, foods that are avoided include raw and uncooked meat, poultry and eggs, unpasteurized milk (raw milk) soft cheeses, fresh fruits and vegetables, raw nuts, raw oats, unpasteurized juices, etc. (Lund and O’Brien, 2011; Lund, 2014; van Dalen et al., 2016). An important concern is that these neutropenic dietary guidelines are set up from a clinical and nutritional perspective, rather than from a food processing and food preservation perspective. It is difficult to unambiguously include or exclude a certain food product since its microbiological contamination is depending on the processing and preservation technologies applied during the manufacturing of the food. In addition, with the increasing number of minimally processed (fresh-like) and new plant-based and hybrid food products set on the market, knowledge of ingredients and applied food technology is not easy to deduce for dieticians providing guidance on a low-microbial diet.

Moreover, the high dietary restrictions can make it difficult for patients to adhere to the diet, and demand increased attention to the nutritional status of the patients, since strict exclusion of certain food groups such as (fresh) fruits and vegetables may lead to unintended reduced nutrient or energy intake (Lund, 2019). The limitations of the neutropenic diet raise the question among healthcare practitioners and dieticians whether it is possible to liberalize or re-orientate the diet by the addition of extra food groups. Especially different plant-based foods, such as dried products (nuts and seeds, herbs, and spices, etc.) and fruit and vegetable juices, are of interest to be included in a neutropenic diet, since they may provide both nutrients and energy to the diet, as well as an increased flavor and taste of self-prepared meals (e.g., by addition of herbs or spices during or post-cooking). Any liberalization should, however, be well thought out and potential food products that would add nutritional or sensorial benefits to include in a neutropenic diet should be assessed with knowledge of food microbiology and preservation technologies. Minimally processed plant-based foods, for instance, are not only intrinsically highly contaminated with (Gram-negative) bacteria, but their microbiological contamination is also highly variable and depends on the technology used during (minimal) processing (Gómez-López et al., 2008; De Corato, 2020). Some of these products may or may not be subjected to a decontamination treatment such as a blanching step, steam-drying, intensive sanitizer treatment/wash before drying, or irradiation, impacting their microbiological contamination (Uyttendaele et al., 2018).

The aim of this study is to critically evaluate the composition of a neutropenic diet, and include experimental research (microbiological lab analysis) regarding plant-based foods, to collect information on their microbiological profile and support the assessment of these plant-based foods as fit-for-purpose in a neutropenic diet. The current guidelines prescribed by the Ghent University Hospital (Ghent, Belgium), indicating which food groups are allowed to be consumed or should be avoided, were revised taking into account the current state of the art of food preservation technologies and good practices during storage and distribution but also including the knowledge available on the expected microbiological profile and standards that apply for these foods according to national guidelines and food business’ microbiological specifications (Uyttendaele et al., 2018). Certain foodstuffs that showed to be difficult to judge were further investigated in detail. Specifically, a selection of (minimally processed) plant-based foodstuffs were sampled on the retail market in Flanders, Belgium and analysed for their microbial contamination to get a better insight into whether these products would indeed be suitable to be included in a neutropenic diet.

## 2. Materials and methods

### 2.1. Evaluation of the current dietary guidelines of a neutropenic diet

The current dietary guidelines prescribed by the Ghent University Hospital (Ghent, Belgium) were evaluated with a focus on reasonably expected microbiological profiles and the state-of-the-art application of food preservation technologies. The evaluation was performed based on the available scientific literature on the type of food processing and intrinsic characteristics of the foods and based upon the authors’ expertise in food microbiology and preservation technologies during a more than 20 years track record of applied research to support microbiological guidelines for a wide range of food categories (dairy, meat, seafood, plant-based foods, bakery products, composite foods, shelf-stable food, and water) and subcategories therein. An applied reference book is Uyttendaele et al. (2018). Where current dietary guidelines for a neutropenic diet for immunocompromised persons were considered inadequate, revised guidelines were proposed.

### 2.2. Microbiological profiling of a selection of plant-based foodstuffs

#### 2.2.1. Selection of foodstuffs and sample collection

The evaluation of the dietary guidelines (Section 2.1) concluded that the microbial contamination of several (minimally processed) plant-based foodstuffs, such as dried fruits, herbs, spices, juices, etc. is highly variable, both intrinsically, as well as depending on the applied processing technologies and the level of good practices applied among different processing companies. These products are therefore difficult to judge as “low microbial contamination”. Nevertheless, from a nutritional point of view, these products might be interesting to include in a neutropenic diet, adding both nutritional and sensorial benefits to the patient’s diet. Therefore, a microbiological screening of various dried, plant-based foodstuffs, as well as pasteurized and freshly squeezed unpasteurized fruit and vegetable juices, was performed. Since it is generally accepted that unprocessed and fresh-cut fruits and vegetables should be avoided in the framework of a neutropenic diet, these products were not included in the screening.

Prepacked dried products and pasteurized juices were purchased from conventional retailers (Delhaize, Colruyt, Albert Heijn, Carrefour, Aldi, Lidl, etc.) in Flanders, Belgium between September 2019 and August 2020 and included dried raisins (*n* = 25), white peppercorns (*n* = 11), ground white pepper (*n* = 19), dried leafy herbs (*n* = 9), peeled (*n* = 5) and unpeeled (*n* = 5) almonds and pasteurized fruit (*n* = 10) and vegetable juices (*n* = 16). Fruit juices included single type as well as mixtures from different types of fruits, whereas all vegetable juices consisted of mixtures of different types of fruits and vegetables. The unpasteurized fruit and vegetable juices (*n* = 16) were either freshly prepared juices, purchased in a takeaway juice bar in Ghent, Belgium (*n* = 6), or self-prepared in the laboratory (*n* = 10) by the use of an automatic juice extractor (Braun, type 4290, Kronberg, Germany).

Dry products were stored according to the instructions on the label (in a dry, cool place) for a maximum of 1 week before analysis. Pasteurized juices were purchased in duplicate and analyzed immediately after purchase as well as after storage at 4°C or 7°C until the end of the shelf-life as indicated on the label. Unpasteurized juices were purchased or prepared in duplicate and analyzed immediately after purchase or preparation, as well as after storage for 24 h at 9°C (reasonable temperature abuse at the consumer stage (Roccato et al., 2017b)) in a closed, glass bottle. The latter (short) storage period was included to simulate next-day consumption after the purchase or preparation of non-prepacked foods.

#### 2.2.2. Physicochemical and microbiological analyses

The pH (edge® pH HI2002-02, Hanna Instruments) of the juice samples was measured on the day of purchase, as well as at the end of shelf-life. Several microbiological parameters were determined (Table 1). Briefly, 10 g portions of each sample were diluted in 90 mL peptone-physiological salt solution (PPS, 8.5 g/L NaCl and 1 g/L neutralized bacteriological peptone (Oxoid, LP0034)) and homogenized for 1 min using a lab stomacher. A serial tenfold dilution in PPS was prepared and appropriate dilutions were plated on the respective agar media, after which plates were incubated according to the conditions in Table 1. To determine spore concentrations, 10 mL of the primary dilution was heated at 80°C for 10 min to eliminate vegetative cells, prior to the preparation of the dilution series. The results were recorded as CFU per gram or milliliter of the sample.

**Table 1:** Microbiological parameters analyzed in the screening of the sampled products [based on Uyttendaele et al. (2018)].

Parameter	Medium	Incubation conditions	Dried products	Juices
Aerobic mesophilic count	PCA (Oxoid, CM0325)	3 d, 30°C	X	X
Aerobic psychrotrophic count	PCA (Oxoid, CM0325)	5 d, 22°C		X
Aerobic spores	PCA (Oxoid, CM0325)	3 d, 30°C		X
Gram-negative bacteria	PCA (Oxoid, CM0325) + 2 mg/L crystal violet (Sigma-Aldrich, C6158)	3 d, 30°C	X	
Yeasts and molds	YGC (Bio-Rad, 3564104)	5 d, 25°C	X	X
Lactic acid bacteria	MRS (Oxoid, CM0361) + 1.4 g/L sorbic acid (Sigma-Aldrich, S1626)	3 d, 30°C	X	X
*E. coli* and coliforms	RAPID’*E.coli* 2 (Bio-Rad, 3564024)	24 h, 37°C		X
*Pseudomonas* spp.	*Pseudomonas* agar base (Oxoid, CM0559) + CFC supplement (Oxoid, SR0103)	48 h, 25°C	X	
*Enterococcus* spp.	SB (Oxoid, CM0377)	48 h, 37°C	X	
*B. cereus*	MYP (Oxoid, CM0929)	24 h, 30°C	X	X
*B. cereus* spores	MYP (Oxoid, CM0929)	24 h, 30°C		X
*L. monocytogenes*	ALOA (Bio-Rad, 3564043)	48 h, 37°C	X	
Coagulase-positive staphylococci	BP (Oxoid, CM1127)	48 h, 37°C	X	

#### 2.2.3. Data processing and statistical analysis

Descriptive statistics were performed on the log-transformed values (mean, standard deviation, 50th, 75th, and 95th percentile, minimum and maximum) in Microsoft Excel 2016. Values below the detection limit were replaced by a value of half of the limit of detection for the calculations (medium bound).

## 3. Results

### 3.1. Evaluation of the dietary guidelines of a neutropenic diet

The current dietary guidelines for a neutropenic diet, prescribed by the Ghent University Hospital, were evaluated. For each currently included and excluded foodstuff, an evaluation was conducted if suitable (or not) for a neutropenic diet, based on scientific evidence of the effect of the current standard use of processing and preservation technologies and their impact on the microbiological quality, safety, and hygiene of the processed foods. Three criteria were found to be important in the evaluation (Table 2). First, the quantitative microbial load of the product was taken into account. A product with a low general microbial contamination (e.g., a low total plate count) does not ensure a safe product but results in a (statistically) lower exposure to (high levels) of (opportunistic) pathogens, and thus in a reduced probability of disease. Further, the composition of the microbiota was taken into account. In this respect, a high concentration of lactic acid bacteria (mainly beneficial bacteria) in, e.g., fermented products was accepted unlike high concentrations of Gram-negative bacteria, the latter a parameter that was used in this study as an indicator for the potential presence of (higher concentrations of) opportunistic pathogens, such as species of *Pseudomonas*, *Serratia*, *Enterobacter*, etc. Secondly, the potential presence of established foodborne pathogens causing disease in the general population (also in lower concentrations), such as *Salmonella* spp., and *Campylobacter coli/jejuni*, was considered by taking into account reported prevalence data of these pathogens in these foods. Lastly, additional focus was put on the potential (post-)contamination of the food with *Listeria monocytogenes*, an opportunistic foodborne pathogen related to a high mortality rate specifically in immunocompromised individuals (Vázquez-Boland et al., 2001). The evaluation and subsequent motivation for revised guidelines for a selection of meat and meat products, dairy products, and plant-based products are shown in Tables 3–5, respectively.

**Table 2:** Three types of foodborne microorganisms taken into account in the evaluation of foodstuffs to be included in or excluded from the guidelines of a neutropenic diet and corresponding motivation.

	Zoonotic agents (*Salmonella* spp., *Campylobacter jejuni/coli*, Shiga toxin-producing *E. coli*)	*Listeria monocytogenes*	Opportunistic pathogens
Risk for the general population	Established foodborne pathogens causing gastrointestinal disease. Typically low infective dose (can be as low as 10 cells for certain strains) (1).	Established foodborne pathogen causing fever, blood poisoning, meningitis, and abortion. Higher infective dose (10^3^–10^6^ cells) compared to zoonotic pathogens (1). Typically mild response in healthy individuals.	Naturally occurring on food (in high concentrations), and thus naturally a part of a consumer’s diet. No evidence of foodborne infections in healthy individuals. Therefore considered to pose a low risk for the general population.
Risk for immuno-compromised patients	Increased severity of disease, probability of complications, hospitalization, and infection-related mortality in immunocompromised individuals (1).	Immunocompromised individuals have a lower resistance to illness and will therefore become ill at lower doses compared to healthy adults. The disease typically has a more severe course and has a high mortality rate in immunocompromised patients.	Several Gram-negative bacteria are known to cause nosocomial infections (2–5). The infective dose is unknown (and depends upon the host), but lowering the dose minimizes the probability of disease. Although Gram-positive bacteria are also identified as opportunistic pathogens, they are also considered beneficial organisms (starter cultures, probiotic properties) and are more likely to have the GRAS status (6–10).
Consideration for suitability in a neutropenic diet	Zoonotic agents are already highly controlled for the general population. GMP and HACCP principles reduce their prevalence. For *Salmonella* spp. and STEC, for instance, a food safety criterion in the EU legislation 2073/2005 states the absence of these agents in 25 g product for several (ready-to-eat) products. These low levels might not be enough for safe consumption in a neutropenic diet. Therefore, an additional zero-tolerance policy could be advised.	Lower concentrations of *L. monocytogenes* in foodstuffs are tolerated for the general population (e.g., in EU legislation 2073/2005: 100 CFU/g at the end of the shelf-life). Additional focus is, however, needed in a neutropenic diet, where these low concentrations cannot be tolerated. A zero-tolerance policy should apply.	Exposure to bacteria should be minimized by applying a low-microbial diet, primarily focusing on Gram-negative bacteria. Consumption of products with lower microbial contamination (and low level of Gram-negative bacteria) results in lower exposure to (opportunistic) pathogens and a reduced probability of disease. The presence of (higher levels of) Gram-positive bacteria can be accepted.

(1) Uyttendaele et al. (2018); (2) Fusco et al. (2018); (3) Bhunia (2018); (4) Lister et al. (2009); (5) Brooke (2012); (6) Ben Braïek and Smaoui (2019); (7) Murphy and Frick (2013); (8) Song et al. (2017); (9) Franz et al. (1999); (10) Kanmani et al. (2013).

**Table 3:** Evaluation and revision of the dietary guidelines of a neutropenic diet related to meat and meat products.

Current guideline	Evaluation	Revised guideline	References
*Products currently allowed in the neutropenic diet*			
Pre-packed salami	- High presence of lactic acid bacteria (typically >10^7^ CFU/g) due to fermentation. Safety of this product is associated with hurdle technology (pH drop, use of salt, ripening period, etc.) and contamination with unwanted microbiota will be depending on the production process. - Industrial production uses starter cultures, leading to rapid acidification and/or production of antimicrobial substances. Artisanal production often relies on spontaneous fermentation from environmental microbiota, being less controlled and thus resulting in a more variable contamination. - Industrial slicing and packaging in a high-care zone reduce the risk for post-contamination with *L. monocytogenes*, compared to non-prepacked products from butcher shops.	Industrially produced and pre-packed salami presents a lower risk compared to artisanal salami, as well as ripened, dried sausages without the use of starter cultures. The latter types of products should be avoided in the framework of a neutropenic diet.	De Boeck et al. (2016); Laranjo et al. (2019); Roccato et al. (2017a)
Pre-packed meat pâté	- Cooked meat product, thus generally has a low level of contamination. However, highly susceptible to post-contamination with *L. monocytogenes* during slicing. - Industrial slicing and packaging in a high-care zone reduce the risk for post-contamination with *L. monocytogenes*, compared to non-prepacked products from butcher shops.	Industrially produced and pre-packed meat pâté presents a lower risk compared to meat pâté from butcher shops.	De Boeck et al. (2016)
Pre-packed deli salads	- Considered a stable product, by the use of hurdle technology (a_w_, pH, acetic acid, and addition of chemical preservatives such as benzoic and sorbic acid). - Industrial packaging in a high-care zone reduces the risk of post-contamination with *L. monocytogenes*, compared to non-prepacked products from butcher shops.	Industrially produced and pre-packed deli salads present a lower risk compared to deli salads from butcher shops.	De Boeck et al. (2016); Vermeulen et al. (2007)
Fully cooked minced meat	- Cooking minced meat ensures sufficient reduction in microbial contamination. - Chicken minced meat (or a preparation thereof), specifically, is highly contaminated with *Campylobacter* spp. Fully cooking (to an internal temperature of 70°C) would be sufficient for the elimination of *Campylobacter* spp., however, a small visual color difference between a semi- and well-cooked product in the case of minced poultry meat (in comparison to, e.g., beef products) makes evaluation of adequate cooking difficult.	Fully cooked minced meat is safe to consume in a neutropenic diet, although minced chicken meat (or preparations thereof) should be avoided.	Lahou et al. (2015); Sampers et al. (2010)

**Table 4:** Evaluation and revision of the dietary guidelines of a neutropenic diet related to dairy products.

Current guideline	Evaluation	Revised guideline	References
*Products currently allowed in the neutropenic diet*			
Pre-packed soft cheeses (fresh cheese, cream cheese, ricotta, mozzarella, feta, etc.) Pre-packed hard cheeses (e.g., Cheddar, Emmental, Parmesan) Ice cream Butter	- Artisanal dairy products, especially those prepared from raw milk, are high-risk products for several foodborne pathogens (*L. monocytogenes*, STEC, *Staphylococcus aureus*, etc.). Pasteurization ensures the inactivation of these vegetative pathogens and leads to lower-risk products. - Specifically for cheeses, post-contamination with *L. monocytogenes* may occur during cutting in deli retail establishments. For dairy products in general, industrial packaging in a high-care zone or in-pack pasteurization ensures a lower risk for post-contamination. - Hard cheeses are considered lower risk compared to soft cheeses. - Surface mold-ripened cheeses or blue cheeses should be avoided.	Industrially produced, pasteurized and preferably pre-packed (not deli retail establishments) dairy products can be consumed in a neutropenic diet. Hard cheeses should be preferred over soft (surface mold-ripened) cheeses.	Lahou and Uyttendaele (2017); Verraes et al. (2015)
*Products currently not allowed in the neutropenic diet*			
“Fresh” herbal cheeses	- “Fresh” cheese has the connotation to be prepared from raw milk but is defined as cheese that is ready for consumption shortly after manufacture (i.e., unripened) and can be produced from both raw and pasteurized milk. - In industrial manufacturing, added herbs typically have undergone a heat (or other) treatment to ensure a 6-log reduction of vegetative pathogens.	Industrially produced herbal cheese poses a low risk and can be allowed in a neutropenic diet. (Artisanal) products with untreated herbs should be avoided.	FAO/WHO (2021) Personal communication with industry

**Table 5:** Evaluation and revision of the dietary guidelines of a neutropenic diet related to fruits, vegetables, and other plant-based products.

Current guideline	Evaluation	Revised guideline	References
*Products currently allowed in the neutropenic diet*			
Frozen vegetables	- Frozen vegetables are only blanched before freezing, which ensures insufficient microbial reduction. Moreover, during the further production process (cooling, freezing, and packaging), frozen vegetables may be post-contaminated with *L. monocytogenes*.	Frozen vegetables should be properly cooked before consumption.	EFSA (2020); PROFEL (2020)
Frozen fruits	- No heat or other treatment resulting in microbial inactivation is included in the processing of frozen fruits, before freezing. The level of contamination of frozen fruits is therefore similar to its fresh counterpart. - Typical for soft red fruits is a high prevalence of Norovirus.	Frozen fruits should be properly cooked before consumption.	De Keuckelaere et al. (2015); Jacxsens et al. (2017)
Cornflakes and other pre-packed breakfast cereals	- Cornflakes are extruded breakfast cereals and have thus been exposed to high temperatures. They, therefore, typically have a low level of contamination. - Muesli is not (necessarily) heat-treated and typically highly contaminated with Gram-negative bacteria.	Only extruded cornflakes can be consumed in a neutropenic diet. Muesli, with or without added nuts, raisins, etc. should be avoided.	Personal communication with industry
*Products currently not included in the neutropenic diet*			
Humus, tapenades, etc.	- Product produced from raw, plant-based ingredients, thus potentially highly contaminated with Gram-negative bacteria. - Ambient stored products are sterilized (in-pack) and therefore typically have a low level of contamination (commercially sterile). Processing of refrigerated products (pasteurization at the most) lacks sufficient inactivation of microorganisms, but rather relies on the cold chain, the addition of additives, and packaging for inhibition of growth and increase of the shelf-life.	Refrigerated types of humus, tapenades, etc. (typically packed in plastic jars) should be avoided. Ambient-stored products (typically packed in glass jars) are safe to consume in a neutropenic diet.	Tuytschaever et al. (2023)

From the evaluation and revised guidelines, it was clear that it is impossible to unambiguously accept or reject a certain type of foodstuff without prior knowledge of the processing and preservation technologies applied during manufacturing and subsequent storage conditions. These technologies largely determine both the quantitative and qualitative microbial contamination of the product and thus its suitability for a neutropenic diet. In this respect, products undergoing a processing step resulting in a (sufficient) reduction in microbial contamination were preferred over raw, unprocessed foodstuffs. Similarly, products produced in an industrial production process were preferred over artisanal products. The former process is considered to be a predictable and standardized production process targeting a decreased contamination with (unwanted) microorganisms by the use of well-implemented and often certified food safety management systems, application of interventions such as a heat inactivation or use of starter cultures in fermentation, proper infrastructure including high-care areas, systematic control on cleaning and disinfection procedures, regular food safety and hygiene training of personnel, etc. Lastly, it was shown that post-contamination with *L. monocytogenes* is important to consider. Also here, industrial processing, e.g., packaging in a high-care zone, extensive control on cleaning and disinfection, etc. reduces the risk for post-contamination significantly.

### 3.2. Microbiological profiling of a selection of plant-based foodstuffs

The microbiological contamination of a selection of dried plant-based foodstuffs and fruit and vegetable juices on the retail market in Flanders, Belgium is shown in Tables 6, 7, respectively. As expected, the microbial contamination of the dry products is highly variable, with aerobic mesophilic counts ranging from values below the detection limit (1 log CFU/g) up to a maximum count of 5.60 log CFU/g for a sample of dried herbs. Of all tested samples, unpeeled almonds seem to be the lowest contaminated with a maximum aerobic mesophilic count of 2.48 log CFU/g. Peeled almonds were, contrary to the expectation, higher contaminated compared to the unpeeled ones but contained a lower concentration of Gram-negative bacteria. Re-analysis showed slightly elevated levels of lactic acid bacteria on the peeled almonds (concentrations of the five samples, respectively, <1, 1.30, 2.04, 2.76, and 2.87 log CFU/g) compared to the unpeeled almonds (four samples <1 log CFU/g, one sample 1.90 log CFU/g). The presence of lactic acid bacteria on the peeled almonds could be the result of post-contamination during the peeling process. Comparing ground white pepper and white peppercorns, it can be noted that although their general contamination level (mesophilic count) is similar, the ground pepper has a lower concentration of Gram-negative bacteria. Dried herbs have the highest contamination of all tested samples, both generally, with a maximum mesophilic count of 5.60 log CFU/g, and with Gram-negative bacteria, with a maximum concentration of 4.05 log CFU/g.

**Table 6:** Microbiological contamination of the sampled dry plant-based products, expressed in log CFU/g.

		Ground white pepper (*n* = 19)	White peppercorns (*n* = 11)	Raisins (*n* = 25)	Dried herbs (*n* = 9)	Unpeeled almonds (*n* = 5)	Peeled almonds (*n* = 5)
Aerobic mesophilic count	Mean	2.54	2.05	3.28	3.01	1.95	2.82
	S.D.	1.24	1.43	0.98	1.45	0.72	0.82
	P50	2.18	1.95	3.11	3.11	2.18	3.08
	P75	3.69	2.32	3.83	3.36	2.34	3.26
	P95	4.11	4.58	4.94	5.04	2.45	3.54
	Min	0.70	0.70	1.60	1.00	0.70	1.48
	Max	4.59	4.84	5.36	5.60	2.48	3.61
Gram-negative bacteria	Mean	0.93	1.54	0.76	2.16	1.33	0.82
	S.D.	0.51	1.02	0.23	0.96	0.74	0.27
	P50	0.70	0.70	0.70	2.11	1.00	0.70
	P75	0.85	2.36	0.70	2.61	2.00	0.70
	P95	1.79	3.03	1.18	3.51	2.20	1.18
	Min	0.70	0.70	0.70	0.70	0.70	0.70
	Max	2.64	3.28	1.70	4.05	2.26	1.30
Yeasts	Mean	1.70	1.73	1.71	N.A.	1.70	2.43
	S.D.	0.00	0.09	0.06	N.A.	0.00	0.70
	P50	1.70	1.70	1.70	N.A.	1.70	2.70
	P75	1.70	1.70	1.70	N.A.	1.70	2.85
	P95	1.70	1.85	1.70	N.A.	1.70	3.15
	Min	1.70	1.70	1.70	N.A.	1.70	1.70
	Max	1.70	2.00	2.00	N.A.	1.70	3.23
Molds	Mean	2.32	2.39	2.68	N.A.	3.30	1.70
	S.D.	0.98	1.14	1.09	N.A.	0.44	0.00
	P50	1.70	1.70	2.48	N.A.	3.00	1.70
	P75	3.00	2.45	3.11	N.A.	3.48	1.70
	P95	4.07	4.58	5.01	N.A.	3.90	1.70
	Min	1.70	1.70	1.70	N.A.	3.00	1.70
	Max	4.70	5.04	5.41	N.A.	4.00	1.70

N.A., not analyzed.

**Table 7:** Microbiological contamination of the sampled fruit and vegetable juices on the day of purchase (D.P.) and at the end of the shelf-life (E.S.), expressed in log CFU/mL.

		Pasteurized fruit juices (*n* = 10)		Pasteurized vegetable juices (*n* = 16)		Unpasteurized juices (*n* = 16)	
		D.P.	E.S.	D.P.	E.S.	D.P.	E.S.
Aerobic mesophilic count	Mean	1.54	2.16	2.35	2.68	5.54	5.39
	S.D.	1.45	2.72	0.64	0.93	0.39	0.45
	P50	2.04	0.95	2.38	2.71	5.42	5.28
	P75	2.77	4.98	2.73	3.21	5.68	5.65
	P95	3.11	5.95	3.27	3.94	6.23	6.20
	Min	−0.30	−0.30	1.46	1.48	5.05	4.68
	Max	3.18	6.02	3.36	5.13	6.36	6.23
Aerobic psychrotrophic count	Mean	1.65	2.11	2.54	2.48	N.A.	N.A.
	S.D.	1.61	2.80	0.64	0.99	N.A.	N.A.
	P50	1.34	1.05	2.43	2.47	N.A.	N.A.
	P75	3.00	4.89	2.84	3.06	N.A.	N.A.
	P95	3.78	5.91	3.44	3.74	N.A.	N.A.
	Min	−0.30	−0.30	1.43	1.08	N.A.	N.A.
	Max	3.84	5.98	4.15	5.05	N.A.	N.A.
Aerobic spores	Mean	0.63	0.49	1.25	1.70	2.05	2.08
	S.D.	1.04	1.39	0.61	0.87	1.15	0.87
	P50	0.35	−0.30	1.41	1.65	2.29	2.14
	P75	1.43	0.71	1.52	2.45	3.13	2.62
	P95	2.06	2.91	2.16	2.81	3.31	3.10
	Min	−0.30	−0.30	0.00	−0.30	−0.30	−0.30
	Max	2.36	3.98	2.20	3.15	3.34	3.40
Yeasts	Mean	1.76	2.23	0.83	0.83	3.98	3.98
	S.D.	1.39	2.51	0.43	0.54	0.51	0.72
	P50	0.85	0.70	0.70	0.70	4.08	4.00
	P75	2.79	3.56	0.70	0.70	4.28	4.66
	P95	3.93	6.44	1.36	1.24	4.70	4.81
	Min	0.70	0.70	0.70	0.70	3.18	2.60
	Max	4.20	6.51	2.43	2.85	4.75	4.88
Molds	Mean	0.91	1.08	0.70	0.72	3.33	3.02
	S.D.	0.68	0.63	0.00	0.08	0.83	1.21
	P50	0.70	0.70	0.70	0.70	3.46	3.38
	P75	0.70	1.45	0.70	0.70	3.72	3.73
	P95	1.88	2.12	0.70	0.77	4.21	4.21
	Min	0.70	0.70	0.70	0.70	0.70	0.70
	Max	2.85	2.18	0.70	1.00	4.30	4.32
Lactic acid bacteria	Mean	0.08	0.06	−0.30	−0.30	3.38	3.32
	S.D.	1.20	1.15	0.00	0.00	1.05	1.14
	P50	−0.30	−0.30	−0.30	−0.30	2.98	2.97
	P75	−0.30	−0.30	−0.30	−0.30	4.45	4.50
	P95	1.79	1.70	−0.30	−0.30	4.69	4.66
	Min	−0.30	−0.30	−0.30	−0.30	1.00	0.70
	Max	3.51	3.34	−0.30	−0.30	4.70	4.72

N.A., not analyzed.

Coagulase-positive staphylococci, *Pseudomonas* spp., enterococci, *L. monocytogenes*, and *B. cereus* were analyzed on a subset of 10 samples of pepper, 11 samples of raisins, 11 samples of dried herbs, and 10 samples of almonds. These specific (opportunistic) human pathogens were either not detected (below 2 log CFU/g) or present in low numbers (max. 3.23 log CFU/g *Pseudomonas* spp. for a sample of dried herbs).

Pasteurized fruit and vegetable juices both generally have a low level of contamination on the day of purchase, with a mesophilic aerobic count ranging from values below the detection limit (0 log CFU/g for liquid samples) up to 3.18 log CFU/mL and 1.46 to 3.36 log CFU/mL, respectively. During the shelf-life, the mesophilic count of most (7/10) fruit juices remained relatively constant or even decreased (concentration ranging from values below detection limit to 2.45 log CFU/mL), while for three samples (mainly containing pineapple, orange, and berries), the mesophilic count increased to a concentration of more than 5 log CFU/mL. Regarding the composition of the microbiota of these fruit juices, the increased mesophilic count was also related to an increased yeast count at the end of the shelf-life. The pasteurized vegetable juices were found to be more stable, with only one sample having an increased mesophilic count of 5.13 log CFU/mL at the end of the shelf-life. All other samples (15/16) had a mesophilic count ranging from 1.48 to 3.54 log CFU/mL. Due to the lack of a pasteurization step, the fresh juices had a higher level of contamination on the day of production or purchase, compared to the pasteurized juices. Regarding the composition, also the number of yeasts, molds, and lactic acid bacteria was shown to be increased compared to the pasteurized juices. *E. coli* was not detected (below 1 log CFU/mL) in any of the juice samples.

The pasteurized juices were also specifically analyzed for *B. cereus*, both on the day of purchase and at the end of the shelf-life. *B. cereus* was not detected (below 1 log CFU/mL) in any of the fruit juice samples on the day of purchase, while 8 out of 16 samples of vegetable juices yielded a positive result, with concentrations ranging from 1.00 to a maximum of 3.34 log CFU/mL on the day of purchase. *B. cereus* was shown to be mostly found in juices prepared from green vegetables (cucumber, spinach, celery, etc.). The concentration was only slightly altered during the shelf-life, with 6 out of 16 positive samples at the end of the shelf-life, having concentrations ranging from 1.00 to a maximum of 3.48 log CFU/mL. Five and 3 out of 16 samples of fresh (unpasteurized) juices were shown positive for *B. cereus* on the day of purchase and at the end of the shelf-life, respectively, with concentrations ranging from 1.00 to 2.60 log CFU/mL and 1.30 to 1.78 log CFU/mL, respectively.

The pH of the juice samples was monitored on the day of purchase, as well as at the end of the shelf-life. The pH of the pasteurized fruit juices (*n* = 10), pasteurized vegetable juices (*n* = 16), and unpasteurized juices (*n* = 16) on the day of purchase ranged between 3.09 and 3.86, 3.42 and 4.66, and 3.12 and 5.17, respectively, and remained relatively constant during the shelf-life (pH range at the end of the shelf-life between 3.10 and 3.85, 3.41 and 4.68, and 3.13 and 5.22, respectively).

## 4. Discussion

### 4.1. Principles and importance of a low-microbial diet

Although the value of a neutropenic diet has been questioned multiple times (van Dalen et al., 2016; Moody et al., 2018; Ball et al., 2019; Sonbol et al., 2019; Taggart et al., 2019; Heng et al., 2020; Ramamoorthy et al., 2020), it is still prescribed to patients with a severe, established immunodeficiency in the day-to-day practice. The present study investigated the principles of a neutropenic diet taking into account current knowledge of food preservation technologies and the expected microbiological profile of different foodstuffs. Three criteria were identified in this study that could be taken into account when evaluating the suitability of a (ready-to-eat) food product to be included in or excluded from a neutropenic diet (Table 2).

First, a ready-to-eat food product should generally have a low level of microbial contamination. Although no outbreak evidence is available linking foodstuffs having a high microbial load or containing specific opportunistic pathogens to the development of (opportunistic) infections in neutropenic patients, historical evidence shows that an increased level of hygiene and successful implementation of good practices (GHP, GMP) lead to lower levels of contamination of foodstuffs, resulting in a better quality of foodstuffs as well as in products that are less likely to contain (increased levels of) pathogens, and thus in less disease (da Cruz et al., 2006; de Oliveira et al., 2016). Furthermore, in developing microbial infections, the dose is considered the main factor influencing both the likelihood of occurrence of symptoms and the severity of the disease (Buchanan et al., 2000; Zwietering and Havelaar, 2006; Haas, 2015). This urges the need to focus on minimizing the exposure to (high concentrations of) microorganisms and Gram-negative bacteria (a parameter that was used in this study as an indicator for the presence of opportunistic pathogens) in particular, which, in its turn, will minimize the likelihood of developing (opportunistic) infections. It is evident that the complete elimination of microorganisms in foods is an unattainable goal, the aim should, therefore, be to include foodstuffs having a concentration of (Gram-negative) bacteria that is as low as reasonably achievable (ALARA).

Secondly, there should be increased vigilance for certain established foodborne pathogens from which it is generally accepted that they are related to a rather moderate risk for the general population (if consumed in low concentrations), but from which the same (low) dose can lead to a more severe response in immunocompromised patients. A typical example is *L. monocytogenes*, which has an increased infective dose compared to zoonotic pathogens but is related to highly severe infections with a high mortality rate in immunocompromised patients (Vázquez-Boland et al., 2001). Implementation of sufficient preventive and intervention strategies during industrial production of foodstuffs such as pasteurization, high-care zones for packaging, extensive control of cleaning and disinfection procedures, etc. minimizes the risk for (post-)contamination of foodstuffs with *L. monocytogenes*.

Thirdly, a zero-tolerance policy should be applied to the established zoonotic pathogens *Salmonella* spp., *Campylobacter coli/jejuni,* and STEC. Due to the impaired immunity and disease state of an immunocompromised patient, any additional infection, and so also foodborne infection, should be avoided. Moreover, they are at increased risk for complications, hospitalization, and mortality resulting from these infections compared to the general population. The already extensive control measures (based on GMP and HACCP principles) applied to these pathogens, because of their relevance to the general population, lead to a low prevalence (ALARA) in foodstuffs. Still, the achieved prevalence does not equal a zero-risk situation (Zwietering et al., 2021) and might, therefore, not be sufficient for immunocompromised patients. An additional zero-tolerance policy could be advised for these established zoonotic pathogens. Such policy may be achieved by the consumption of industrially produced foods as well as the implementation of additional control measures at the consumer stage, such as a cooking step.

A combination of these three criteria creates a clear framework for evaluation of the suitability of (newly developed) foodstuffs to be included in or excluded from a neutropenic diet. It also shows, despite the increasing criticism, the necessity of a neutropenic diet to reduce the risk of foodborne infections in immunocompromised patients. Not only does the implementation of a neutropenic diet decrease the risk for any (opportunistic) infection, but it also decreases the risk for specific foodborne infections caused by the consumption of ready-to-eat foodstuffs inherently (highly) contaminated with foodborne pathogens. Especially the latter cannot be controlled by safe food handling alone, but can only be controlled by excluding certain ready-to-eat products in the framework of a neutropenic diet (Lund, 2019). From the perspective of this newly developed framework, however, preference should be given to naming the diet a “low-microbial diet” instead of a “neutropenic diet” for increased clarity in communication among both healthcare practitioners and dieticians, and patients.

### 4.2. Impact of processing technologies on the microbial contamination of plant-based foods

Due to consumer preferences shifting towards foods with more natural and fresh-like sensorial properties, different plant-based foodstuffs and ingredients are nowadays set to the market as foods that are minimally processed. In the strict sense, minimally processed vegetables are any fresh vegetables that have been physically altered from their original form but remain in a fresh state (Gómez-López et al., 2008). These foods typically only have undergone one or more of the following actions: cleaning, coring, peeling, chopping, slicing or dicing, washing (with or without sanitizers), freezing, mashing, and unpasteurized juicing or blending (EFSA, 2013; Uyttendaele et al., 2018). In the broader sense, minimal processing may also comprise other (new) processing technologies that are regarded as milder alternatives to the conventional food preservation processes based on heat treatment (pasteurization and sterilization), such as (gentle) drying, high-pressure processing, etc. The variety of technologies implemented in the processing of plant-based foods has, however, a profound impact on the microbial contamination of the final product.

Most processes included in the strict definition of minimal processing are physical interventions and typically have no or only a limited impact on the microbial composition of the product (Uyttendaele et al., 2018). Typical examples of these minimally processed plant-based foods are fresh-cut vegetables, pre-cut fruit mixes, unpasteurized juices, frozen fruits and vegetables, etc. The microbial safety of these products is not guaranteed by aiming at microbial inactivation during processing, but rather by the prevention of contamination by relying on good practices and HACCP principles and inhibition or delay of growth (and thus prolongation of the shelf-life) by maintaining the cold chain. Despite being sufficient for the general population, the applied production process is not enough to rely on for a “zero-risk” situation. Due to the absence of a microbial inactivation step, these minimally processed products are still contaminated with (high concentrations of) Gram-negative bacteria and might still contain established foodborne pathogens. They should therefore be generally avoided in the framework of a neutropenic diet.

Drying technologies are primarily designed and evaluated from a technological perspective, i.e., based on their performance to reduce the water activity at a low energy cost, and as such, accomplish long-term stability, while the exposure to elevated temperatures is usually not validated as a critical control point to ensure a satisfactory level of microbial reduction (Bourdoux et al., 2016). In practice, dried products are mainly produced by sun or solar drying (in (small) farms in developing countries) or conventional air drying (in an industrial setting) (Schweiggert et al., 2007). In the latter, large varieties of processing temperatures are used, resulting in a varying degree of microbial inactivation. However, to preserve product quality and minimize organoleptic changes during the drying process, often gentle drying conditions are applied and thus microbial reduction will be limited (Bourdoux et al., 2016). To increase the inactivation of microorganisms, several additional decontamination methods such as steam treatment or irradiation may or may not be applied during processing (Schweiggert et al., 2007). As a result, dried products are produced using a whole variety of drying technologies and may thus also have a varying degree of microbial contamination, which was also observed in the microbiological screening (Table 6).

High-pressure processing (HPP) is a relatively new, nonthermal food processing technology, often applied to fruit and vegetable juices as an alternative to classical thermal pasteurization. The technology minimizes the effect on the sensorial and nutritional quality of the product and as such retains the characteristics of fresh, “minimally processed” foods having superior quality and nutritional value compared to thermally processed foods (Considine et al., 2008). HPP is an effective microbial inactivation technology, from which the rate of inactivation depends on different factors such as the magnitude of the pressure and the holding time (Considine et al., 2008; Podolak et al., 2020). Considering the current lack of a broad safe-harbor process that covers varying juice products, producers themselves should conduct validation studies to ensure the process will deliver a minimum of 5-log reduction of the pertinent microorganism (Podolak et al., 2020). In general, it can be concluded that pasteurized juices, regardless of whether pasteurization was performed thermally or using high pressure, will have a lower microbial contamination, compared to unpasteurized juices (Table 7).

Intensive thermal processing (pasteurization or sterilization) is still the most traditional and most effective food preservation technology to eliminate foodborne pathogens. However, an increasing number of alternative (minimal) processes are being applied to plant-based foods, resulting in a large variety of microbial contamination of these products. For certain products, no microbial inactivation is achieved in the production process (the strict sense of minimal processing), for others, inactivation is an intrinsic property of the technique (HPP), and for some products, the degree of inactivation is variable (drying). Because of this large variety in applied processing technologies, knowledge of ingredients, applied processing technology, and the resulting level of microbial contamination is not easy to be deduced for providing guidance on a low-microbial diet. Furthermore, this information is not present on the label of a food product, therefore, consumers are unable to judge a product’s suitability for inclusion in a low-microbial diet.

### 4.3. Microbiological profiling and evaluation of (minimally processed) plant-based foodstuffs

As explained in the previous paragraph, the microbial contamination of several (minimally processed) plant-based foodstuffs is highly variable because of the large variety of applied processing technologies. Microbiological analyses were performed to get insight into their general and specific microbial contamination. The subsequent evaluation of the different foodstuffs to be included in a neutropenic diet was investigated based on the three criteria described in paragraph 4.1. Regarding the general microbial contamination (total count and Gram-negative bacteria), a low concentration was defined as approximately 2–3 log CFU/g, where products would preferably have a total count below 3 log CFU/g and a concentration of Gram-negative bacteria below 2 log CFU/g. Within a product category, the 95th percentile (P_95_) of the determined parameter was used for the evaluation towards the proposed safety limits, corresponding to a 5% probability of randomly selecting a product with a higher concentration in the supermarket. However, not only the microbiological status is important to be considered in the evaluation of a foodstuff, but also nutritional and sensorial properties should be taken into account. The proposed microbiological limits were therefore not regarded as “fixed rules”, an attempt was made to find a balance between (microbiological) risks and (nutritional) benefits of consuming certain products.

It was shown that the microbial contamination of the **dried products** (pepper, raisins, and dried herbs) was highly variable, as expected from the production process (Section 4.2). The P_95_ of the aerobic mesophilic count of these products consistently exceeded 4 log CFU/g, therefore, these products should be avoided in the framework of a neutropenic diet. Taking into account the small portion size, (preferably ground) **pepper** could be an exception. Considering a maximum observed concentration of 4.59 log CFU/g on ground white pepper and a portion size of a few 100 mg of pepper on a total product of a few 100 g, the addition of pepper would only contribute to an additional contamination of about 1–2 log CFU/g in the final product. Furthermore, pepper is also important for sensorial reasons, especially for immunocompromised patients with a reduced taste and appetite. It should be noted that pepper (and other spices as well as herbs) could be efficiently decontaminated using gamma-irradiation, resulting in products with a distinct lower contamination level (Uyttendaele et al., 2018). Although the method is efficient, safe, and allowed in the EU, it is generally not well-accepted by consumers (Schweiggert et al., 2007; EFSA, 2011a,b). Because of this negative risk perception, these irradiated products are (unfortunately) not for sale as B2C product on the Belgian market.

Contrary to the expectation, the peeled **almonds** showed to have a higher level of contamination compared to the unpeeled almonds, exceeding the established safety limit of 3 log CFU/g. Looking into the composition of the microbiota of these products, it was shown to mostly consist of lactic acid bacteria and yeasts, probably due to a post-contamination after the peeling process. On the other hand, the concentration of Gram-negative bacteria was lower in the peeled almonds, below the proposed safety limit. Therefore, the lowest risk can be expected in roasted, peeled almonds, which can thus be safely consumed as an energy-rich, nutritious snack.

The category of **fruit and vegetable juices** is a diverse product group both on the level of ingredients and the level of processing conditions. Although both factors influence the microbial contamination of the end product, the results showed that the inclusion of a pasteurization step, rather than the selection of ingredients was the major determinant of the suitability of these juices in a neutropenic diet. An industrial pasteurization step not only reduces the general level of contamination but is also specifically focused on the elimination of (vegetative) foodborne pathogens. Pasteurized juices are therefore considered the lower-risk alternative to fresh juices and can safely be incorporated into a neutropenic diet, possibly serving as a nutritious alternative to fresh fruits and vegetables. Unpasteurized juices, especially those from takeaway juice bars, should be avoided since there is no prior knowledge of the level of hygienic practices applied during production. Self-prepared juices could be considered safe, if they are produced from thickly peeled (e.g., oranges, apples, cucumber, etc. but not berries, grapes, spinach, etc.) or pre-cooked fruits and/or vegetables. Next-day consumption of self-prepared juices is, however, not recommended. Although the results showed a constant total count during the 24-h storage, still foodborne pathogens might be present (due to the lack of a pasteurization step) and might proliferate in the juice during the short storage period in the case of elevated pH values (five of ten samples of self-prepared juices were shown to have a pH higher than 4.4 in this study). Finally, also the ingredients influence the microbial contamination of the juices. Green vegetable juices were shown to harbor (slightly) elevated levels of *B. cereus* (max. Concentration of 3.34 log CFU/g). These elevated levels may be present as natural contaminants or residues of the use of *B. thuringiensis* biopesticides during primary production (Rosenquist et al., 2005; Frederiksen et al., 2006; Frentzel et al., 2020; Zhao et al., 2021). As *B. thuringiensis* is considered an opportunistic pathogen, it might be better to avoid (green) vegetable juices and thus fruit juices might be preferred.

In general, the sample numbers in this microbial screening were low. In the end, the analyses were performed mostly to show, based on the authors’ track record of experience, the variability of microbial contamination, and the difficulty of “judging” a product’s suitability of inclusion in a low-microbial diet. Furthermore, no analyses for the presence of primary foodborne pathogens such as *Salmonella* spp. or Shiga toxin-producing *E. coli* were performed. Especially dried products are known to be occasionally linked to *Salmonella* spp. since this pathogen is known to survive well under conditions of low water activity (dry foods; Zweifel and Stephan, 2012). The prevalence of these pathogens in these dry products is, however, known to be (very) low (Table 8), while for the juices, the inclusion of a pasteurization step ensures the elimination of these vegetative pathogens. Still, as shown in Table 8, the prevalence never equals zero and a residual risk remains. This residual risk could be further reduced by the implementation of appropriate hygienic measures at the consumer stage, such as roasting of nuts.

**Table 8:** Occurrence of *Salmonella* spp. in food categories in EU from 2016 to 2020: percentage positive result (number of samples).

	**2016**	**2017**	**2018**	**2019**	**2020**
Fruits, vegetables, and juices	0.12 (4,309)	0.80 (6,497)	0.38 (8,126)	0.07 (7,007)	0.08 (7,931)
Herbs and spices	1.51 (1,390)	0.42 (2,631)	0.90 (2,440)	0.33 (2,136)	0.83 (1,561)
Nuts and cereals	No data	No data	No data	0.23 (436)	0.08 (1,330)

EFSA (2017, 2018, 2019, 2021a,b).

### 4.4. Guidance for food hygiene, handling, and processing

The inclusion or exclusion of certain foodstuffs in a low-microbial diet is one thing, still, implementation of general hygienic practices and safe food handling is at least as important. Dietary guidelines and hygienic measures should be regarded as complementary in a low-microbial diet to decrease the exposure of immunocompromised consumers to microorganisms. Therefore, the application of the WHO’s Five Keys to Safer Food (keep clean, separate raw and cooked, cook thoroughly, keep food at safe temperatures, and use safe water and raw materials; WHO, 2006) during preparation and storage of foodstuffs is important, not only to avoid additional contamination during preparation but also to avoid bacterial toxin formation (e.g., due to temperature abuse). Additionally, the stricter rule “cook it, peel it, or forget it” is supported as a simple message that all can understand.

Furthermore, from a microbiological point of view, preference should be given to foodstuffs produced in an industrial production process, which is considered a stable process aimed at lower microbial contamination. From a nutritional point of view, however, often the opposite is true and preference is given to unprocessed foods since these are often regarded as “healthy” foods (e.g., fresh fruits and vegetables). This leads to the discussion of avoiding (ultra-)processed foods for nutritional reasons versus preferring processed foods for microbiological reasons. Therefore, multidisciplinary research is needed in which a balance between the different disciplines should be found.

## 5. Conclusion

The neutropenic diet is currently based on nutritional and clinical knowledge, often without taking food microbiology or food processing and preservation technologies into account. Differences in processing technologies, initial contamination of products, etc., lead, however, to a high degree of variability in microbial contamination and makes it difficult to assess the suitability of a product in the framework of a neutropenic diet. Complete elimination of microorganisms in foods is unattainable, but lowering the dose to which patients are exposed, lowers the probability of developing disease. Finally, food research requires multidisciplinary communication and collaboration, taking into account nutritional, microbiological, and technological properties, leading to healthy, nutritional, and safe foods for immunocompromised consumers.

## Data availability statement

The original contributions presented in the study are included in the article/supplementary material, further inquiries can be directed to the corresponding author.

## Author contributions

TB performed the experiments and data analysis, and prepared the manuscript. All authors contributed to the discussions leading to the proposed revision of the neutropenic diet, in which FM, SM, and MR provided clinical and dietary expertise, and TB, LJ, and MU provided expertise in food microbiology. MU and LJ conceived and designed the study and revised the manuscript. All authors contributed to the article and approved the submitted version.

## Funding

TB is supported as a research assistant by Ghent University.

## Conflict of interest

The authors declare that the research was conducted in the absence of any commercial or financial relationships that could be construed as a potential conflict of interest.

## Publisher’s note

All claims expressed in this article are solely those of the authors and do not necessarily represent those of their affiliated organizations, or those of the publisher, the editors and the reviewers. Any product that may be evaluated in this article, or claim that may be made by its manufacturer, is not guaranteed or endorsed by the publisher.
